# Correlation of Oxidative Stress Parameters and Inflammatory Markers in Tunisian Coronary Artery Disease Patients

**Published:** 2011-03

**Authors:** Azza Dandana, Imen Gammoudi, Salima Ferchichi, Hinda Chahed, Hlima Ben Limam, Faouzi Addad, Abdelhedi Miled

**Affiliations:** 1 *Department of Clinical Biochemistry, CHU-Farhat Hached, Sousse, Tunisia;*; 2 *Department of Cardiology, CHU-Fattouma Bourguiba Monastir, Tunisia*

**Keywords:** glutathione peroxidase (GPX), total antioxidant status (TAS), high sensitivity C reactive protein (hs-CRP), cardiovascular diseases, diabetes

## Abstract

**Introduction:**

Oxidative stress is now recognized as being the cause and the consequence of cardiovascular diseases.

**Objective:**

The role that oxidative stress parameters and inflammatory markers may play in diabetes and related cardiovascular disease (CVD) among Tunisian coronary diabetic patients.

**Patients and methods:**

We measured the erythrocyte glutathione peroxidase (GPX), the superoxide dismutase (SOD) activities and the plasmatic total antioxidant status (TAS) concentration by colorimetric methods, the hs-CRP by immunonephelometry assays.

**Results:**

TAS and GPX were significantly decreased among patients compared to the controls (1.14 ± 0.28 mmol/l vs 1.55 ± 0.35 mmol/l; 59.32 ± 10.72 U/gHb vs 149.19 ± 30.95 U/gHb). For the diabetic patients, TAS is correlated positively with hs-CRP (r=0.01, *p*<10^-3^). At the not diabetic subjects, TAS is correlated negatively with the hsCRP.

**Conclusion:**

Determination of antioxidative defense markers contributes to understanding the effect of stress oxidative on the development and the prevention of cardiovascular disease.

## INTRODUCTION

Although mortality from Cardiovascular Diseases (CVD) has shown a tendency to decrease in developed countries, and it still remains the main cause of death.

The origins of these diseases are multifactorial, but some of the important causes may be attributed to cardiovascular risk factors (CVRF). Age and gender, are unmodifiable CVRF whereas smoking, high blood pressure, hypercholesterolemia …are modifiable CVRF (1).

Diabetes mellitus (DM) have also been shown to play an important role in CVD (1). Patients with diabetes have a two, to threefold increased incidence of CVD and those who are present in the fourth and fifth decade of life have a twofold increase in mortality (2). Actually, interest has developed in alternative markers, such as plasma markers of oxidative stress which have a role to predict CVD risk (3).

However, oxidative stress plays a big role in the development of many pathologies.

In fact hyperglycaemia can increase oxidative stress through several pathways and induce intracellular Reactive Oxygen Species (ROS) produced by the proton electrochemical gradient generated by mitochondrial electron transport chain and resulting in increased production of superoxide (4). The other mechanism involves the transition metal catalysed auto-oxidation of free glucose yielding superoxide anion and hydrogen peroxide (5).

There is also evidence that hyperglycaemia may comprise natural antioxidant defense. Under normal circumstances free radicals are rapidly eliminated by antioxidants such as reduced glutathione, vitamin C and vitamin E. Reduced glutathione content, as well as reduced vitamin E, have been reported among diabetics patients (6).

But now days, we know that there were additional participants in diabetic development that involves inflammation, endothelial injury, lipoproteins retention in the arterial wall, and lipoproteins modifications (7). Hyperglycaemia can promote vascular complications by multiple postulated mechanisms. Increased glucose concentrations can activate nuclear factor-κB (8), a key mediator that regulates multiple pro-inflammatory and pro-atherosclerotic target genes in endothelial cells (ECs), vascular smooth muscle cells (VSMCs), and macrophages (7). Inflammation has been strongly implicated in both atherosclerosis and type 2 DM (8, 9). Despite this, no single mechanism has been suggested why this pattern is found in diabetic patients (10). C-reactive protein (CRP), a hepatically derived marker of systemic inflammation, is the prototypic inflammatory biomarker. A direct role for CRP in the pathogenesis of vascular damage has been proposed and is supported by some, but not all, experimental data (11). CRP is independently associated with incident CVD and is the only biomarker to be endorsed in guidelines for primary prevention (12, 13).

So, its exploration seems to be interesting to study the implications of the CVD and the diabetes. The purpose in this study is to explore the antioxidant status by determining the SOD, GPX and the total antioxidant status (TAS) among coronary diabetic patients and by evaluating inflammation by using high sensitivity C reactive protein (hs-CRP).

## PATIENTS AND METHODS

### Study population

The study population consist of two groups. The control subjects are 120 healthy volunteers (80 men and 40 women) with no history of CVD, diabetes or cerebrovascular diseases. Their mean age is 40 ± 7 years.

150 consecutive patients (115 men and 35 women) with angiographically documented CVD were enrolled from Cardiovascular Department of University Hospital Fattouma Bourguiba of Monastir, Tunisia. The mean age of this group is 61.15 ± 10.64 years.

This group of patients made of 70 diabetic and 80 non diabetic. All participants were interviewed, and data in dyslipidemia, DM, hypertension, smoking habits were recorded.

### Determination of antioxidant parameters

SOD, GPX activities and TAS concentration were performed using commercials tests manufactured by Randox Laboratories (UK, Antrium) in a Daytona – analyser.

### Laboratory analysis

Serum total cholesterol (TC), triglycerides (TG), high density lipoprotein cholesterol (HDL-C) and glucose were measured with colorimetric assays using an automated system (Cx 9 Pro-Bechman Coulter – Fuller –Ton, CA).

In Addition, blood samples were collected for the determination of serum apolipoproteins (ApoA1 and ApoB), high sensitive C-reactive proteins (hs-CRP) and fibrinogen according to the instructions of the manufacture using particle-enhancer immunonephelometric assays (BNII Dade Behring, Marburg, Germany).

### Statistical analysis

Statistical analysis was done with *Spss 15.0*. Testing of numerical characteristics was done by using *Student’s t-test*, and testing of correlations by use of *Pearson’s test*. All parameters were given as (mean ± SD).

## RESULTS

Characteristics laboratory variables and the values of antioxidative parameters patients and controls subjects are shown in Table 1.

**Table 1:** Characteristics laboratory variables and the values of antioxidative parameters patients and controls subjects

		Patients (n=150)	Controls (n=120)	

Age	**(years, X ± δ)**	61.23 ± 10.81	40 ± 7	ns
BMI	**(Kg/m^2^, X ± δ)**	28.51 ± 5.02	27.8 ± 3.4	ns
Diabetes	**(%)**	46.7	0	
Hypertension	**(%)**	48	0	
Smoking	**(%)**	48	35	
GPX	**(U/gHb, X ± δ)**	59.32 ± 10.72	140.19 ± 30.95	*P*<0.001
TAS	**(mmol/l, X ± δ)**	1.14 ± 0.28	1.55 ± 0.28	*P*<0.001
SOD	**(U/gHb, X ± δ)**	629.58 ± 392.033	1378.47 ± 360.95	*P*<0.001
Glucose	**(mmol/l, X ± δ)**	8,8 ± 3,92	4.50 ± 1.8	*P*<0.001
ApoA1	**(g/l, X ± δ)**	1.21 ± 0.98	1.55 ± 0.28	*P*<0.001
ApoB	**(g/l, X ± δ)**	1.45 ± 0.49	0.86 ± 0.22	*P*<0.001
ApoB/ApoA1		1.85 ± 1.45	0.57 ± 0.19	*P*<0.001
hs-CRP	**(mg/l, X ± δ)**	7.6 ± 8.8	2.09 ± 0.34	*P*<0.001
Fibrinogen	**(g/l, X ± δ)**	3.9 ± 1.39	2.61 ± 0.51	*P*<0.001
HDL-C	**(mmol/l, X ± δ)**	1.3 ± 0.45	1 ± 0.37	*P*<0.001
LDL-C	**(mmol/l, X ± δ)**	3.75 ± 0.98	1.88 ± 0.8	*P*<0.001
TG	**(mmol/l, X ± δ)**	1.8 ± 1.15	0.97 ± 0.28	*P*<0.001
TC	**(mmol/l, X ± δ)**	5.97 ± 1.12	3.6 ± 0.45	*P*<0.001

Statistical data processing still significantly lower TAS, GPX, SOD and hs-CRP values among patients compared to the controls (TAS: 1.14 ± 0.28 mmol/l vs 1.55 ± 0.355 mmol/l, GPX: 59.32 ± 10.72 U/gHb vs 149.19 ± 30.95 U/gHb, SOD: 629.58 ± 392.033 U/gHb vs1378.47 ± 360.95 U/gHb, hs-CRP: 7.6 ± 8.8 mg/l vs 2.09 ± 0.34 mg/l) (Table 1).

Table 2 shows characteristics laboratory variables and the values of antioxidative parameters in diabetic patients and none diabetic subjects. Diabetic patients had significantly lower values of TAS, GPX, SOD and and hs-CRP in relations with coronary subjects without diabetics complications (TAS: 1.15 ± 0.25 mmol/l vs 1.14 ± 0.29 mmol/l, GPX: 58.37 ± 10.63 U/gHb vs 60.28 ± 10.70 U/gHb, SOD: 615.42 ± 396.97 U/gHb vs 651.23 ± 389.76 U/gHb, hs-CRP: 7.94 ± 10.82 mg/l vs 7.5 ± 6.6 mg/l). Additional, coronary patients with diabetic complications had significantly higher values of ApoB/A1 ratio, fibrinogen and TG compared to non diabetic patients (Table 2).

**Table 2:** Characteristics laboratory variables and the values of antioxidative parameters in diabetics patients and non diabetics subjects

		Diabetics patients (n=70)	Non diabetics patients (n=80)	

Age	**(years, X ± δ)**	62.26 ± 9.63	60.48 ± 11.52	ns
BMI	**(Kg/m^2^, X ± δ)**	29.40 ± 4.87	27.87 ± 5.08	ns
GPX	**(U/gHb, X ± δ)**	58.37 ± 10.63	60.24 ± 10.70	*P*<0.001
TAS	**(mmol/l, X ± δ)**	1.15 ± 0.27	1.14 ± 0.29	*P*<0.001
SOD	**(U/gHb, X ± δ)**	615.42 ± 396.97	651.23 ± 389.76	*P*<0.001
Glucose	**(mmol/l, X ± δ)**	9.6 ± 4.22	5.01 ± 1.4	*P*<0.001
ApoA1	**(g/l, X ± δ)**	1.36 ± 1.10	1.09 ± 0.82	*P*<0.001
ApoB	**(g/l, X ± δ)**	1.41 ± 0.48	1.47 ± 0.5	*P*<0.001
ApoB/ApoA1		1.55 ± 1.09	2.08 ± 1.65	*P*<0.001
hs-CRP	**(mg/l, X ± δ)**	7.94 ± 10.82	7.5 ± 6.6	*P*<0.001
Fibrinogen	**(g/l, X ± δ)**	4.07 ± 1.34	3.9 ± 1.43	*P*<0.001
HDL-C	**(mmol/l, X ± δ)**	1.05 ± 0.35	1.07 ± 0.41	*P*<0.001
LDL-C	**(mmol/l, X ± δ)**	3.53 ± 1.9	3.44 ± 1.09	*P*<0.001
TG	**(mmol/l, X ± δ)**	2 ± 1.23	1.67 ± 1.04	*P*<0.001
TC	**(mmol/l, X ± δ)**	4.25 ± 1.28	4.17 ± 0.9	*P*<0.001

Table 3 shows the correlations between TAS activity and some clinical and laboratory variables in coronary diabetic patients as well as coronary non diabetic subjects.

**Table 3:** Correlations of TAS activity with some clinical and laboratory variables in coronary diabetics patients as well as coronary non diabetics subjects

	Diabetics patients		Non diabetics patients	
	r	p	r	p

Age	-0.033	0.0001	0.017	0.0001
BMI	0.37	0.0001	0.045	0.0001
GPX	0.081	0.0001	0.026	0.0001
SOD	-0.321	0.0001	0.006	0.0001
Glucose	-0.102	0.0001	0.224	0.0001
ApoA1	0.266	0.531 ns	0.179	0.105 ns
ApoB	-0.053	0.0001	0.06	0.0001
ApoB/ApoA1	-0.111	0.0001	-0.04	0.0001
hs-CRP	-0.76	0.0001	0.01	0.0001
Fibrinogen	-0.025	0.0001	0.135	0.0001
HDL-C	-0.121	0.0001	-0.003	0.0001
LDL-C	0.152	0.0001	0.201	0.0001
TG	0.011	0.0001	0.164	0.0001
TC	-0.135	0.0001	-0.105	0.0001

**Figure 1:**
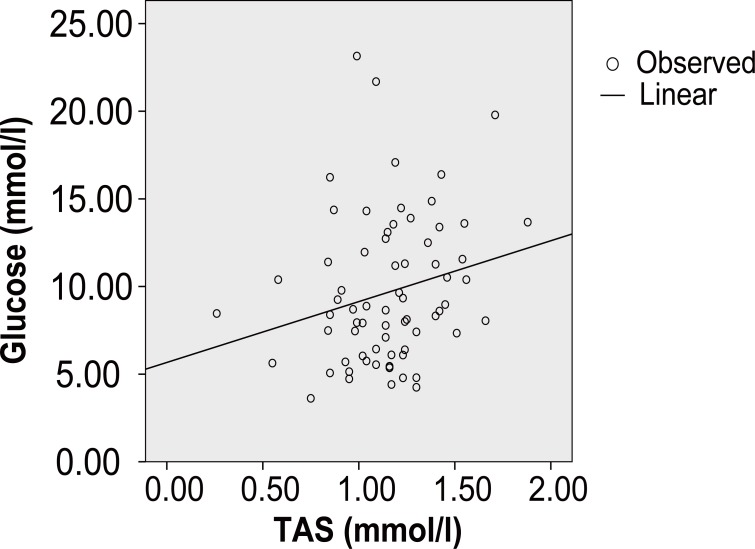
In coronary diabetic patients, Pearson’s correlation coefficient revealed significant positive correlation between TAS and glucose (r=0,224, *p*<10^-3^).

**Figure 2:**
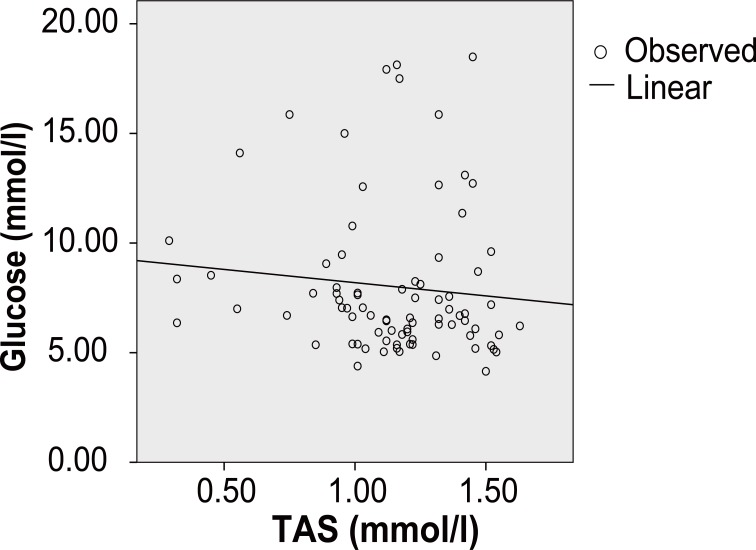
In the coronary none diabetic subjects, the TAS presents a significant negative correlation with glucose (r=-0.102, *p*<10^-3^).

In coronary diabetic patients, *Pearson’s* correlation coefficient shows significant positive correlation between TAS and hs-CRP (r=0.001, *p*<10^-3^) (Figure 1), TAS and ApoB (r=0.06, *p*<10^-3^), TAS and fibrinogen (r=0.135, *p*<10^-3^) and TAS and glucose (r=0.224, *p*<10^-3^) (Figure 1). In coronary none diabetic subjects, the TAS presents a significant negative correlation with the hs-CRP (r=-0.76, *p*<10^-3^), ApoB(r=-0.053, *p*<10^-3^), fibrinogen (r=-0.148, *p*<10^-3^) and glucose (r=-0.102, *p*<10^-3^) (Figure 2).

**Figure 3:**
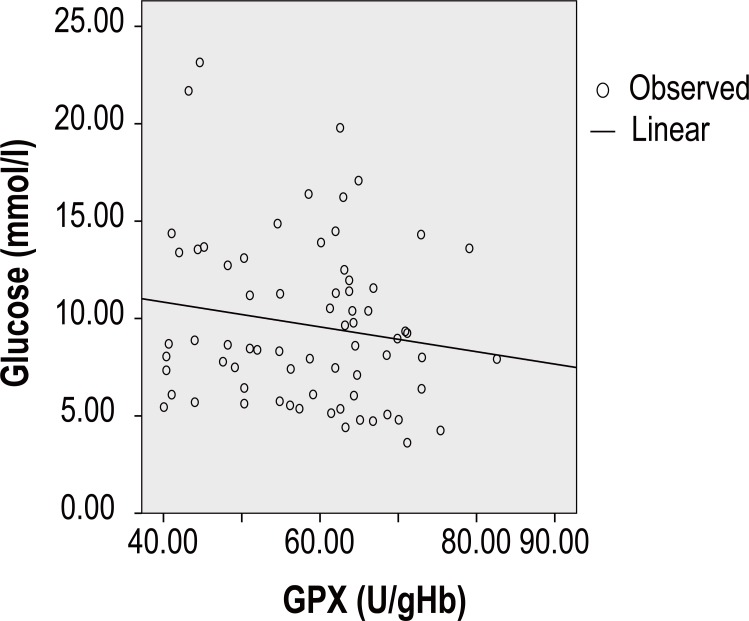
Coronary patients with diabetic complications had significantly negative correlation between GPX-glucose (r=-0.160, *p*<10^-3^).

Then, Table 4 shows the correlations of GPX activity with some clinical and laboratory variables in coronary diabetic patients as well as coronary none diabetic subjects. Coronary patients with diabetic complications had significantly negative correlation between GPX-hs-CRP (r=-0.073, *p*<10^-3^), GPX-glucose (r=-0.160, *p*<10^-3^) (Figure 3) and GPX-fibrinogen (r=-0.149, *p*<10^-3^). In these diabetic patients Pearson’s correlation presents significant positive correlation between GPX and ApoB (r=0.064, *p*<10^-3^).

**Table 4:** Correlations of GPX activity with some clinical and laboratory variables in coronary diabetics patients as well as coronary non diabetics subjects

	Diabetics patients		Non diabetics patients	
	r	p	r	p

Age	-0.031	0.916 ns	0.04	0.0001
BMI	-0.136	0.0001	0.2	0.0001
TAS	0.081	0.0001	0.026	0.0001
SOD	0.021	0.0001	0.076	0.0001
Glucose	0.129	0.0001	-0.16	0.0001
ApoA1	-0.052	0.0001	-0.161	0.0001
ApoB	0.051	0.0001	0.064	0.0001
ApoB/ApoA1	0.109	0.0001	0.198	0.0001
hs-CRP	-0.027	0.0001	-0.073	0.0001
Fibrinogen	-0.145	0.0001	-0.149	0.0001
HDL-C	0.101	0.0001	0.027	0.0001
LDL-C	0.206	0.0001	-0.071	0.0001
TG	0.072	0.0001	-0.153	0.0001
TC	0.015	0.0001	-0.024	0.0001

**Figure 4:**
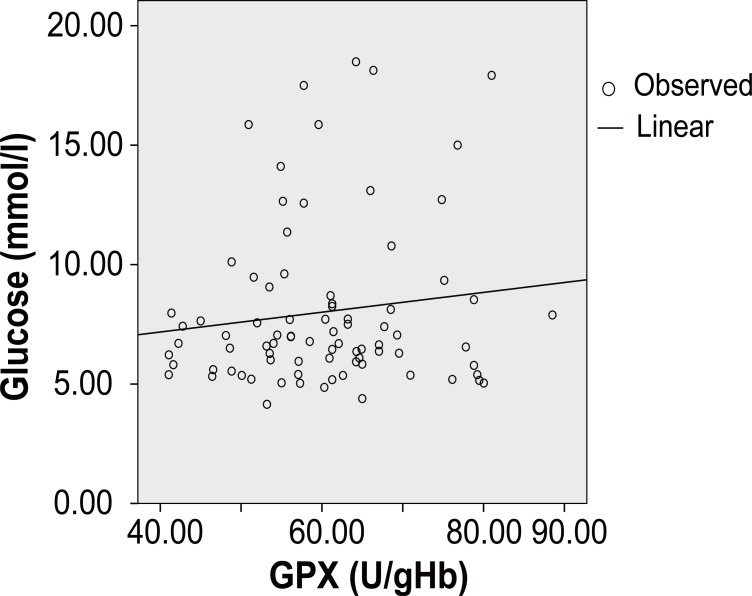
In coronary patients without diabetic complications, Pearson’s correlation revealed a positive significantly correlation between GPX-glucose (r=0.129, *p*<10^-3^).

In coronary none diabetic patients, Pearson’s correlation reveals a positive significantly correlation between GPX-glucose (r=0.129, *p*<10^-3^) (Figure 4), GPX-ApoB (r=0.051, *p*<10^-3^), and a significantly negative correlation between GPX-hs-CRP (r=-0.027, *p*<10^-3^).

**Figure 5:**
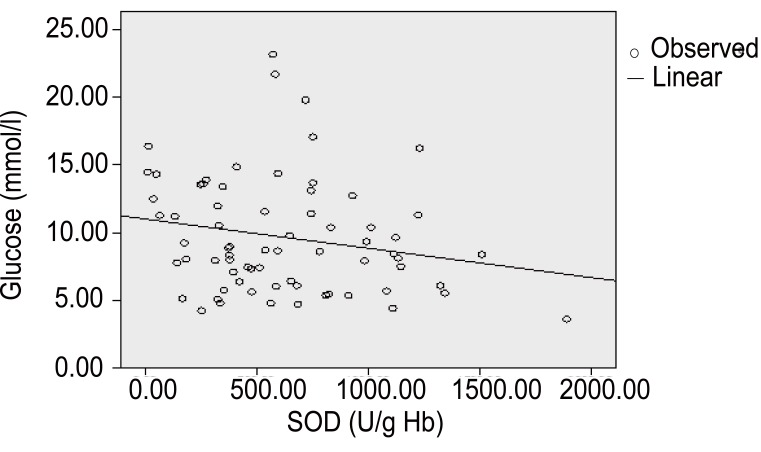
Coronary patients with diabetic complications had significantly negative correlation between SOD-glucose (r=-0.202, *p*<10^-3^).

Moreover, Table 5 shows the correlations between SOD activity and some clinical and laboratory variables in coronary diabetic patients as well as coronary non diabetic subjects. In coronary diabetic patients, *Pearson’s* correlation coefficient shows significant positive correlation between SOD and GPX (r=0.021, *p*<10^-3^). In these diabetic patients Pearson’s correlation presents significant negative correlation between SOD-glucose (r=-0.202, *p*<10^-3^) (Figure 5), SOD-ApoB (r=-0.078, *p*<10^-3^), SOD-hs-CRP (r=-0.167, *p*<10^-3^), and SOD-fibrinogen (r=-0.131, *p*<10^-3^) (Table 5).

**Table 5:** Correlations of SOD activity with some clinical and laboratory variables in coronary diabetics patients as well as coronary non diabetics subjects

	Diabetics patients		Non diabetics patients	
	r	p	r	p

Age	-0.200	0.0001	0.001	0.0001
BMI	-0.095	0.0001	0.040	0.0001
GPX	0.021	0.0001	0.076	0.0001
TAS	-0.321	0.0001	0.006	0.0001
Glucose	-0.202	0.0001	0.112	0.0001
ApoA1	-0.015	0.0001	-0.153	0.0001
ApoB	-0.078	0.0001	0.074	0.0001
ApoB/ApoA1	-0.004	0.0001	0.125	0.0001
hs-CRP	-0.167	0.0001	-0.013	0.0001
Fibrinogen	-0.131	0.0001	-0.119	0.0001
HDL-C	-0.108	0.0001	-0.016	0.0001
LDL-C	-0.187	0.0001	0.181	0.0001
TG	0.064	0.0001	0.089	0.0001
TC	-0.097	0.0001	0.064	0.0001

**Figure 6:**
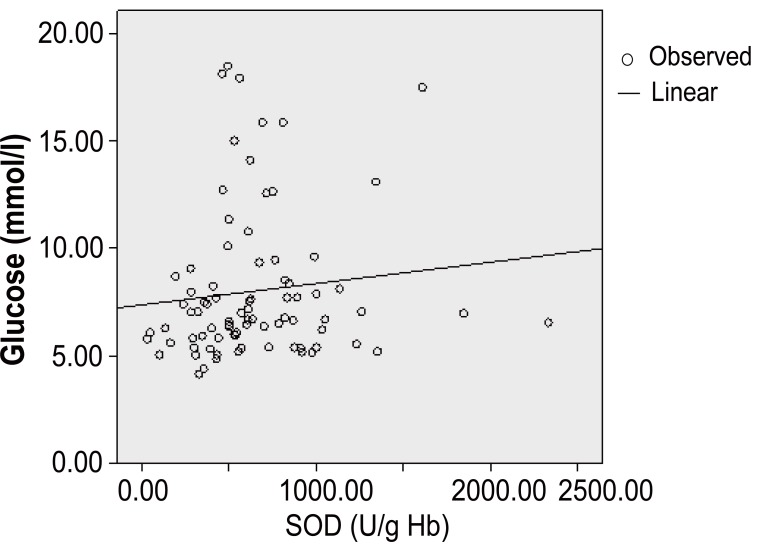
In coronary patients without diabetic complications, Pearson’s correlation revealed a positive significantly correlation between SOD-glucose (r=0,112, *p*<10^-3^).

Although, in coronary none diabetic subjects, the SOD presents a significant positive correlation with the glucose (r=0.112, *p*<10^-3^) (Figure 6) and ApoB (r=0.074, *p*<10^-3^) and a significantly negative correlation between SOD-hs-CRP (r=-0,013, *p*<10^-3^) and SOD-fibrinogen(r=-0.119, *p*<10^-3^) (Table 5).

While, in these two groups, Pearson’s correlation shows a significantly positive correlation between GPX-TAS and GPX-SOD (Table 5).

Finally, Table 6 shows the correlations of hs-CRP activity with some clinical and laboratory variables in coronary diabetic patients as well as coronary non diabetic subjects.

**Table 6:** Correlations of hs-CRP activity with some clinical and laboratory variables in coronary diabetics patients as well as coronary non diabetics subjects

	Diabetics patients		Non diabetics patients	
	r	p	r	p

Age	0.036	0.0001	0.002	0.0001
BMI	0.194	0.0001	-0.141	0.0001
GPX	-0.073	0.0001	-0.027	0.0001
TAS	0.010	0.0001	-0.076	0.0001
SOD	-0.167	0.0001	-0.013	0.0001
Glucose	-0.058	ns	-0.013	ns
ApoA1	0.012	0.0001	0.067	0.0001
ApoB	0.056	0.0001	-0.134	0.0001
ApoB/ApoA1	-0.053	0.0001	-0.170	0.0001
Fibrinogen	0.415	0.003	0.410	0.0001
HDL-C	0.064	0.0001	-0.048	0.0001
LDL-C	-0.073	0.001	-0.149	0.0001
TG	0.141	0.0001	-0.031	0.0001
TC	0.074	0.006	0.091	0.0001

Coronary patients with diabetics complications show significantly positive correlation between hs-CRP-ApoB (r=0.056, *p*<10^-3^), hs-CRP-fibrinogen (r=0.415, *p*<10^-3^) and hs-CRP-TG (r=0.141, *p*<10^-3^) (Table 6).

Although, at the coronary none diabetic subjects, the hs-CRP presents a significant negative correlation with the ApoB (r=-0.134, *p*<10^-3^) and the TG (r=-0.031, *p*<10^-3^) (Table 6).

## DISCUSSION

Diabetes mellitus is a complex and multifactorial disease indulging severe insulin dysfunction in conjunction with gross abnormalities in glucose homeostasis, lipid and protein metabolism. The metabolic dysregulation associated with diabetes causes secondary pathophysiologic changes in multiple organ systems that impose a heavy burden of morbidity and mortality from macrovascular and microvascular complications. Oxidative stress plays an important role in chronic complications of diabetes and is postulated to be associated with increased lipid peroxidation (14). The present study has examined the changes in both extra and intracellular antioxidants status in diabetic patients. Diabetes has been shown to be associated with numerous thrombotic, atherosclerotic, and cardiovascular diseases. Cholesterol has been singled out as the cause of atherosclerosis. However, other lipids, such as triglycerides and phospholipids, also have shown similar correlations (15). In our study, the levels of serum lipids were found to be elevated in diabetic patients. The marked hyperlipemia that characterizes the diabetic state may therefore be regarded as a consequence of the uninhibited actions of lipolytic hormones on fat depots. The increase and fall in the individual lipoprotein levels is a reflection of the total serum cholesterol levels; that is, the levels of VLDL-C, LDL-C, and HDL-C increase or decrease with the level of total serum cholesterol, and it is their ratio that determines the pathophysiology of lipoprotein metabolism (16, 17).

Moreover, many studies have demonstrated the presence of oxidative stress in CVD as an expression of increased free radical production and decreased antioxidant defense (1, 18-20). Also, there are many different markers that can be used to prove the presence of oxidative stress in CVD or in DM (21). Free radicals are very unstable due to their high reactivity (22, 23).

Antioxidants constitute the foremost defense system that limit the toxicity associated with free radicals. The levels of these defense mechanisms are altered in diabetes and, therefore, the ineffective scavenging of free radicals plays a crucial role in determining the extent of tissue injury (24).

Because of their nature, they have a short lifetime and are difficult to measure and accurately determine in vivo as well as in biological material such as plasma or other body fluids. So, on the basis of the obtained results, it may be concluded that the values of studied antioxidative parameters (SOD, GPX and TAS) were significantly lower in coronary patients with or without diabetic complications comparatively with controls.

It is important to highlight that, in the diabetic group the increase of glucose concentration is followed by higher activity of TAS and GPX. This means that among these patients hyperglycaemia induce a positive response from the antioxidative defense system (25).

On the contrary, in coronary none diabetic patients, negative response of antioxidative defense system may be related to the effect of protein glycosylation and the impact of oxidative stress on reduced catalytic SOD, TAS and GPX activity all contributing to impaired total antioxidative defense of diabetics patients with cardiovascular complications (26).

Therefore, it is believed that these enzymes are significant in conditions of oxidative stress in atherosclerotic lesions. GPX is an essential enzyme for the elimination of organic and inorganic peroxides, and it is a crucial intracellular antioxidative enzyme (27).

SOD is considered one of the primary enzymes since they are involved in the direct elimination of ROS. The activity of SOD was found to be lower in diabetic subjects. The observed decrease in SOD activity could result from inactivation by H_2_O_2_ or by glycation of the enzyme, which have been reported to occur in diabetes (28). Therefore, increased oxidative stress may not only result from hyperglycaemia associated with diabetes, but may also have an important causal role in β-cell failure and the development of insulin resistance and type 2 DM (3). Several studies have demonstrated that plasma markers of oxidative stress are elevated in CVD or in the presence of its classical risk factors (29-31).

On the other hand, several studies have suggested that atherosclerosis is a chronic inflammatory disease (32, 33). CRP, an acute phase protein produced mainly in the liver, is an indicator of inflammation. Therefore, patients with type 2 diabetes who are, usually obese, could potentially have high CRP. It is shown also that patients with an elevated CRP have up to 8.5-fold increase in morbidity and mortality (34, 35). In diabetic patients, clustering of traditional and non-traditional CVD risk factors in the same individual may explain their associations with CVD via the common pathway of chronic inflammation. We have studied the associations of hs-CRP concentrations in coronary patients with and without type 2 diabetes. Concentrations of hs-CRP increased when compared with age and gender-matched diabetic controls without CVD suggesting that hs-CRP is a stronger discriminator for detection of CVD in the patients. The elevated hs-CRP concentration may be a reflection of the low level, chronic inflammatory state caused by tissue damage in atheromatous lesions or a consequence of infection with atherogenic organisms such as Helicobacter pylori, Chlamydia pneumoniae, cytomegalovirus or herpes simplex virus (36-38).

Finally, determination of markers of antioxidative defense as very sensitive parameters not only contributes to a better understanding of oxidative stress effect but on CVD development, diabetes and the treatment of these two diseases. Also, as the pathogenesis of both diabetes and cardiovascular disease involves oxidative stress, the use of antioxidants is an appealing therapy (39, 40). Importantly, pharmacologic agents currently in use that have been shown to be effective in reducing cardiovascular mortality are known to have antioxidant properties.

## CONCLUSION

CVD is the major cause of mortality in patients with diabetes, and hense the cost and clinical implications of the condition are significant. Oxidative cellular damage provides a possible explanation for the increase risk seen in diabetes and perhaps for unexplained cardiovascular events in subjects with little in the way of other risk factors. The early initiation of therapy aimed at reducing oxidative stress and/or modulating ROS-sensitive signalling pathways may be of benefit for reducing cardiovascular disease in diabetes. Further insights into the molecular mechanisms of the metabolic basis of diabetes will prove invaluable in the treatment and prevention of this debilitating condition.

## References

[1] Godin DV, Wohaeib SA, Garnett ME, Goumeniouk AD (1988). Antioxidant enzyme alterations in experimental and clinical diabetes. Mol. Cell Biochem.

[2] Panzram G (1987). Mortality and survival in type 2 (non-insulin-dependent) diabetes mellitus. Diabetologia.

[3] Bucala R, Mitchell R, Arnold K, Innerarity T (1995). Identification of the major site of apolipoprotein B modification by advanced glycosylation end products blocking uptake by the low density lipoprotein receptor. J. Biol. Chem.

[4] Touyz RM (2004). Reactive oxygen species, vascular oxidative stress and redox signaling in hypertension: what is the clinical significance?. Hypertension.

[5] Stephens WJ, Khanolkar MP, Bain SC (2009). The biological relevance and measurement of plasma markers of oxidative stress in diabetes and cardiovascular disease. Atherosclerosis.

[6] Nishikawa T, Edelstein D, Du XL, Yamagishi S (2000). Normalizing mitochondrial superoxide production blocks three pathways of hyperglycaemic damage. Nature.

[7] Wolf SP (1993). Diabetes mellitus and free radicals. Free radical transition metals and oxidative stress in the aethiology of diabetes mellitus and complications. Br. Med. Bull.

[8] Orasanu G, Plutzky J (2009). The Pathologic Continuum of Diabetic Vascular Disease. J. Am. Coll. Cardiol.

[9] Hansson GK (2005). Inflammation, atherosclerosis, and coronary artery disease. N. Engl. J. Med.

[10] Shoelson SE, Lee J, Goldfine AB (2006). Inflammation and insulin resistance. J. Clin. Invest.

[11] Dasu MR, Devaraj S, Jialal I (2007). High glucose induces IL-1 beta expression in human monocytes: mechanistic insights. Am. J. Physiol. Endocrinol. Metab.

[12] Kistorp C, Raymond I, Pedersen F, Gustafsson F (2005). N-terminal pro-brain natriuretic peptide, Creactive protein, and urinary albumin levels as predictors of mortality and cardiovascular events in older adults. J. Am. Med. Assoc.

[13] Pasupathi P, Chandrasekar V, Kumar US (2009). Evaluation of oxidative stress, enzymatic and non-enzymatic antioxidants and metabolic thyroid hormone status in patients with diabetes mellitus. Diabetes & Metabolic Syndrome: Diabetes Metab Syndr.

[14] Venkateswaran S, Pari L, Saravanan G (2002). Effect of Phaseolus vulgaris on circulatory antioxidants and lipids in rats with streptozotocin-induced diabetes. J. Med. Food.

[15] Suryawanshi NP, Bhutey AK, Nagdeote AN, Jadhav AA (2006). Study of lipid peroxide and lipid profile in diabetes mellitus. Indian J. Clin. Biochem.

[16] Giugliano D, Acampora R, D’Onofrio F (1994). Medical hypothesis: cardiovascular complications of diabetes Mellitus – from glucose to insulin and back. Diabete Metab.

[17] Noda Y, Mori A, Packer L (1997). Gliclazide scavangers hydroxyl, superoxide and nitric oxide radicals: an ESR study. Mol. Pathol. Pharmacol.

[18] Richard C, O’Brien T, Ming L (1997). The effect of gliclazide and other sulfonylureas on low-density lipoprotein oxidation *in vitro*. Metabolism.

[19] Banga JD (1994). Lower extremity arterial disease in diabetes mellitus. Diabetes Rev. Int.

[20] Paolisso G, Giugliano D (1996). Oxidative stress and insulin action: is there a relationship?. Diabetologia.

[21] Halliwell B, Gutteridge JMC (1989). Free radicals in biology and medicine.

[22] Kuyvenhoven JP, Meinders AE (1999). Oxidative stress and diabetes mellitus pathogenesis of long-term complications. EJIM.

[23] Ramachandran B, Ravi K, Narayanan V, Kandaswamy M (2004). Effect of macrocyclic binuclear oxovanadium complex on tissue defense system in streptozotocin-induced diabetic rats. Clin. Chim. Acta.

[24] Granic P, Bradamente V, Lackovic Z (2001). Laboratorijski pokazatelji okidacijskog stresa. Oksidativni stres i djelotvornost antioksidansa.

[25] Jandric-Balen M, Bozikov V, Bozikov J, Metelko Z (2004). Impact of glycemic control on antioxidant enzyme activity in patients with type 2 diabetes mellitus. Diabetolo Croatica.

[26] Blankenberg S, Rupprecht HJ, Bickel C, Torzewski M (2003). Glutathione peroxidase 1 activity and cardiovascular events in patients with coronary artery disease. N. Engl. J. Med.

[27] Suarna C, Dean RT, May J, Stocker R (1995). Human atherosclerotic plaques contain both oxidized lipids and relatively large amounts of a-tocopherol and ascorbate. Arterioscler Thromb. Vasc. Biol.

[28] Sozmen EY, Sozmen B, Delen Y, Onat T (2001). Catalase/superoxide dismutase (SOD) and catalase/paraoxonase (PON) ratios may implicate poor glycemic control. Arch. Med. Res.

[29] Harrison D, Griendling KK, Landmesser U, Hornig B (2003). Role of oxidative stress in atherosclerosis. Am. J. Cardiol.

[30] Evans JL, Goldfine ID, Maddux BA, Grodsky GM (2002). Oxidative stress and stress-activated signaling pathways: a unifying hypothesis of type 2 diabetes. Endocr Rev.

[31] Suzuki YJ, Forman HJ, Sevanian A (1997). Oxidants as stimulators of signal transduction. Free Radic. Biol. Med.

[32] Liuzzo G, Biasucci LM, Gallimore JR, Grillo RL (1994). The prognostic value of C-reactive protein and serum amyloid a protein in severe unstable angina. New Engl. J. Med.

[33] Ridker PM, Hennekens CH, Buring JE, Rifai N (2000). C Reactive protein and other markers of inflammation in the prediction of cardiovascular disease in women. New Engl. J. Med.

[34] Ross R (1993). The pathogenesis of atherosclerosis: a perspective for the 1990s. Nature.

[35] Patel P, Mendall MA, Carrington D, Strachan DP (1995). Association of Helicobacter pylori and Chlamydia pneumoniae infections with coronary heart disease and cardiovascular risk factors. BMJ.

[36] Mendall MA, Patel P, Ballam L, Strachan D (1996). C reactive protein and its relation to cardiovascular risk factors: a population based cross sectional study. BMJ.

[37] Heart Protection Study Collaborative (2002). MRC/BHF heart protection study of antioxidant vitamin supplementation in 20,536 high-risk individuals: a randomised placebo-controlled trial. Lancet.

[38] Lonn E, Yusuf S, Hoogwerf B, Pogue J (2002). Effects of vitamin E on cardiovascular and microvascular outcomes in high-risk patients with diabetes: results of the HOPE study and MICRO-HOPE substudy. Diabetes Care.

[39] Griendling KK, FitzGerald GA (2003). Oxidative stress and cardiovascular injury. Part II. Animal and human studies. Circulation.

[40] Reilly M, Delanty N, Lawson JA, FitzGerald GA (1996). Modulation of oxidant stress *in vivo* in chronic cigarette smokers. Circulation.

